# Supplementary material for: Investigating the causal effect of maternal continuum of care on child’s minimum acceptable diet: A multilevel approach using partially pooled propensity score weighting
Source: PLoS One. 2025 Nov 10;20(11):e0335972. doi: 10.1371/journal.pone.0335972 (PMC12599957; doi:10.1371/journal.pone.0335972)
Supplement: Attachment — Submitted filename: Response to Editor.pdf [file pone.0335972.s001.pdf]

**To**

**The Editor,**

**PLOS ONE**

Dear Editor,

Thank you very much for your time and allowing us to submit our manuscript titled **“**Investigating the Causal Effect of Maternal Continuum of Care on Child's Minimum Acceptable Diet: A Multilevel Approach using Partially Pooled Propensity Score Weighting” in your reputed journal. We have tried our best to address all of the Journal requirements accordingly.

Sincerely yours,

Mr. Shafayet Khan Shafee
Institute of Statistical Research and Training

////////////////////////////////////////////////////////////////////////////////////////////////////////

**Journal Requirements:**

1. It has come to our attention that the manuscript seems to be a resubmission of the manuscript PONE-D-25-12906, which was previously considered by the journal. It is a requirement by the journal that any manuscript resubmitted after a previous round of review is accompanied by a rebuttal letter outlining the response to the original reviewers’ comments, so that the editors can establish whether the original concerns have been addressed.

**Response: Thank you for your message regarding our submission (PONE-D-25-28053). We would like to clarify that while this manuscript was recognized as a resubmission of an earlier version (PONE-D-25-12906), the previous submission was returned without formal peer review due to the unavailability of an Academic Editor and reviewers, as stated in the decision letter from Editor-in-Chief. Since no reviewer comments were provided, there were no specific concerns to address. While the core content remains unchanged, we have revised the title to better reflect the methodological focus "Partially Pooled Propensity Score Weighting". Given that the previous manuscript did not undergo peer review, we respectfully request that this be treated as a new submission and considered for peer review.**
