# Supplementary material for: Investigating the causal effect of maternal continuum of care on child’s minimum acceptable diet: A multilevel approach using partially pooled propensity score weighting
Source: PLoS One. 2025 Nov 10;20(11):e0335972. doi: 10.1371/journal.pone.0335972 (PMC12599957; doi:10.1371/journal.pone.0335972)
Supplement: Attachment — Submitted filename: Response to Reviewers.pdf [file pone.0335972.s002.pdf]

To
The Academic Editor,
PLOS ONE

Dear Editor,

Thank you for giving us the opportunity to submit a revised version of our manuscript titled *"Investigating the causal effect of maternal continuum of care on child's minimum acceptable diet: A multilevel approach using partially pooled propensity score weighting"* for consideration in PLOS ONE. We sincerely appreciate the time and effort that you and the reviewers have invested in evaluating our work. We are grateful for the constructive comments and insightful suggestions, which have helped us to improve the clarity and quality of the manuscript.

In addition to addressing the reviewer comments, we have also revised the manuscript to ensure compliance with the journal's formatting and submission requirements. All changes are highlighted in the revised manuscript with track changes. Please find below our detailed, point-by-point responses to each comment and concerns. In this response letter, the comments and concerns are highlighted in green for clarity. All page and line numbers mentioned refer to the revised version of the manuscript with track changes.

Sincerely yours,
Shafayet Khan Shafee
Institute of Statistical Research and Training,
University of Dhaka, Dhaka.

Journal Requirements

When submitting your revision, we need you to address these additional requirements,

1. Please ensure that your manuscript meets PLOS ONE's style requirements, including those for file naming. The PLOS ONE style templates can be found at,
   i) https://journals.plos.org/plosone/s/file?id=wjVg/PLOSOne_formatting_sample_main_body.pdf and
   ii) https://journals.plos.org/plosone/s/file?id=ba62/PLOSOne_formatting_sample_title_authors_affiliations.pdf

Author Response: Thank you for pointing this out. We have carefully revised the manuscript to ensure compliance with the PLOS ONE style and formatting requirements. We have also incorporated the updated LaTeX template (verson 3.7, August 2025) provided by PLOS ONE; previously, we were using the version 3.5.

In line with the journal's guidelines, we have:

- Converted the title, section, and subsection headings to sentence-case.
- Removed zip codes from the author affiliations.
- Applied double spacing throughout the manuscript.
- Used "Fig" and "Eq" consistently in place of "Figure" and "Equation" to cite figures and equations.
- Formatted figure titles in bold type.
- Generated all figures in TIFF format in accordance with PLOS ONE's requirements for file dimensions and resolution (following from https://journals.plos.org/plosone/s/figures#loc-figure-file-requirements).
- Updated figure file names to "Fig1.tif", "Fig2.tif", and so on.
- Replaced the previous BibTeX style file (`plos2015.bst`) with the updated `plos2025.bst` provided in the new template, ensuring that the bibliography style now matches the current PLOS ONE standards.
- Made all in-text citations hyperlink to their corresponding references in the bibliography

We hope that these revisions adequately address the journal's formatting standards.

2. Thank you for uploading your study's underlying data set. Unfortunately, the repository you have noted in your Data Availability statement does not qualify as an acceptable data repository according to PLOS's standards.

At this time, please upload the minimal data set necessary to replicate your study's findings to a stable, public repository (such as figshare or Dryad) and provide us with the relevant URLs, DOIs, or accession numbers that may be used to access these data. For a list of recommended repositories and additional information on PLOS standards for data deposition, please see https://journals.plos.org/plosone/s/recommended-repositories.

Author Response: Thank you for this suggestion. Following your recommendation, we have uploaded the minimal datasets necessary to replicate the study findings to a private Figshare repository, which is a PLOS ONE recommended repository. The repository can be accessed via the following link: https://figshare.com/s/0b512877056adf1fdd30.

We have also updated the "Data Availability Statement" (page 17 of the revised manuscript with track changes) to include the DOI of this Figshare repository. Please note that the DOI is not yet

active, as the repository is currently private. The repository will be made publicly accessible upon acceptance/publication of the manuscript.

3. If the reviewer comments include a recommendation to cite specific previously published works, please review and evaluate these publications to determine whether they are relevant and should be cited. There is no requirement to cite these works unless the editor has indicated otherwise.

   Author Response: There were no specific recommendations from the reviewers to cite additional previously published works.

4. Please review your reference list to ensure that it is complete and correct. If you have cited papers that have been retracted, please include the rationale for doing so in the manuscript text, or remove these references and replace them with relevant current references. Any changes to the reference list should be mentioned in the rebuttal letter that accompanies your revised manuscript. If you need to cite a retracted article, indicate the article's retracted status in the References list and also include a citation and full reference for the retraction notice.

   Author Response: Thank you for this suggestion. We have carefully reviewed our reference list to ensure that all citations are complete, accurate, and appropriate. We have also verified that none of the cited works in the revised manuscript have been retracted. During this review process, we have made a few adjustments and additions to our reference list.

   - **Addition of reference [1]:** Used in Section "1. Introduction" (page 3, lines 38, 41, and 47 of the revised manuscript with track changes) to provide the definitions of MMF, milk feeds, and MAD. This cited document specifically presents these indicator definitions in greater detail and with clear organization. It is now listed as the 10th reference in the "References" section of the revised manuscript.
   - **Replacement of a non-peer-reviewed citation:** One of the originally cited references [2], a poster paper presented at the IUSSP XXVIII International Population Conference in Cape Town, South Africa, was not a formally published article. It was originally cited in the sentence:

     > "The healthcare services a woman receives throughout the continuum of maternal care during pregnancy, childbirth, and the immediate postnatal period are vital for the survival and well-being of both the mother and the child"

     on page 4, line 58 of the revised manuscript with track changes. To ensure the citation reflects a peer-reviewed source, we have replaced this with [3], which offers robust, multi-country evidence supporting the importance of the continuum of maternal care for maternal and child health outcomes.

The newly added reference reinforces the statement more strongly and aligns with current literature standards.

- **Addition of reference [4]:** Added in Subsection "2.2 Variables" (page 7, line 189 of the revised manuscript with track changes) to cite the definition of a "Skilled Birth Attendant". This is now the 30th reference in the "References" section of the revised manuscript.
- **Addition of references to support policy action discussion:** In Subsection "4.1 Discussion" (page 15, lines 356–379 of the revised manuscript with track changes), we have added references [5–12] to substantiate the discussion on policy actions for enhancing CoC achievement. They are now listed as references 41 through 48 in the "References" section of the revised manuscript.
- **Addition of references on recall bias:** In Subsection "4.2 Limitations" (page 16, line 387 of the revised manuscript with track changes), we have added references [13, 14] to support the statement that estimates derived from cross-sectional survey data may be subject to recall bias. These are now listed as 49th and 50th references in the "References" section.

In summary, we have replaced one non-peer-reviewed reference and incorporated additional authoritative sources to further strengthen the manuscript.

5. While revising your submission, please upload your figure files to the Preflight Analysis and Conversion Engine (PACE) digital diagnostic tool, https://pacev2.apexcovantage.com/. PACE helps ensure that figures meet PLOS requirements. To use PACE, you must first register as a user. Registration is free. Then, login and navigate to the UPLOAD tab, where you will find detailed instructions on how to use the tool. If you encounter any issues or have any questions when using PACE, please email PLOS at figures@plos.org. Please note that Supporting Information files do not need this step.

Author Response: In accordance with the journal's requirements, we have uploaded all analysis figures to the PACE. The figures were found to be compliant with PLOS ONE standards. PACE only converted the file extensions from `.tiff` to `.tif`. We have attached the PACE-tested figures in our revised submission.

Additional Editor Comments

1. Could you provide details on how the survey data were collected, for example, was this a once off survey? Were the repeated rounds of data collection etc.?. I couldn't figure this out in your methods section as it currently stands. I also struggled to see how you infer "causality" on what seems to me was a cross-sectional survey, hence the comment for you to provide details on the design of the "survey"/study.

   Author Response: Thank you for raising this point. In the earlier version of the manuscript, we lacked sufficient clarification regarding the survey data collection and the rationale for drawing causal inferences from cross-sectional survey data. We appreciate the opportunity to clarify these in the revised manuscripts.

   To clarify the survey data collection method, we now specify in the subsection "2.1 Data Source" (page 6, lines 123–129 of the revised manuscript with track changes) that the MICS 2019 is a cross-sectional household survey conducted in a single round, with no repeated follow-ups.

   In addition, to address potential concerns regarding inferring causality from a cross-sectional survey, we have added text in the "Introduction" section (page 5, lines 113–118 of the revised manuscript with track changes) explaining how the definitions of CoC completion (during pregnancy, delivery, and the immediate postnatal period) and MAD intake (measured among children aged 6–23 months) imply a temporal ordering. This ordering helps establish the causal direction between exposure and outcome. The sequence of maternal care preceding child feeding mitigates the risk of reverse causation and supports the plausibility of causal inference based on cross-sectional data.

   Furthermore, We have already described the use of multilevel inverse probability weighting (IPW) methods to estimate causal effects from observational data. Briefly, IPW uses observed confounders to calculate the probability of exposure (the propensity score) and then reweights individuals so that the exposed and non-exposed groups become comparable on those covariates. This process creates a pseudo-population in which the distribution of confounders is balanced across groups, approximating what would be expected under random assignment [15]. By reducing confounding bias in this way, IPW allows us to draw credible causal inferences from cross-sectional survey data.

   We believe these revisions rectify the earlier omission, clearly specify that the data are cross-sectional with no repeated follow-ups, and provide a well-justified rationale for examining causal effects using cross-sectional survey data.

Reviewers' Comments to the Authors

Reviewer 1

Well done on your manuscript. I am not well versed on the statistical analysis used in the study hence I said, 'I don't know' to the question 'Has the statistical analysis been performed appropriately and rigorously?'

Author Response: Thank you!

Introduction

1. Specify whether the associations you mention here are positive or negative, don't leave the reader guessing for e.g. is the influence of social media, parity, maternal age etc. on CoC and MAD a positive or negative one?

   Author Response: Thank you for this suggestion. In the Introduction, our aim was to highlight the established associations between factors such as social media exposure, parity, maternal age, etc. with both CoC completion and MAD intake, in order to justify their inclusion as potential confounders in our analysis. Because our focus was on illustrating these variables as part of the associational pathway, rather than on the orientation (positive or negative) of their effects [16], we did not specify the direction of the associations at that stage. The statistical adjustment in our analysis addresses these factors irrespective of the sign of their association, ensuring that potential confounding bias is accounted for.

   Accordingly, we have not modified the "Introduction" in this respect, as the emphasis remains on the existence of associations rather than their orientation. We hope this explanation clarifies our rationale and intention for maintaining this presentation.

Variables

2. What is a skilled provider or skilled birth attendant

   Author Response: Thank you for asking this question. The earlier version of the manuscript did not provide a clear definition of skilled providers and skilled birth attendants. In the revised manuscript, we have now added these definitions in the subsection "2.2 Variables" (page 8, lines 182–189 of the revised manuscript with track changes).

   Specifically, skilled providers in this context refer to health professionals such as medical doctors, nurses, midwives, paramedics/medical assistants, family welfare visitors, and community-based skilled birth attendants [17]. A skilled birth attendant is defined as a doctor, nurse, or midwife with accredited

training who is competent to manage normal pregnancies, childbirth, and the immediate postnatal period, as well as to recognize, manage, and refer complications affecting mothers and newborns [4].

3. What are milk foods?

Author Response: Thank you for raising this question. In the original manuscript, the definition of milk feeds was not explicitly described. In the revised manuscript, we have now added this definition in the “Introduction” section (page 3, lines 38–41 of the revised manuscript with track changes).

Specifically, milk feeds refer to any formula (e.g., infant formula, follow-on formula, toddler milk) or any animal milk other than human breast milk (e.g., cow’s milk, goat’s milk, evaporated or reconstituted powdered milk), as well as yogurt and other fermented milk products in semi-solid or drinkable form [1].

Sensitivity analysis

4. Second sentence -‘the approach introduced by?’ Please revise this sentence.

Author Response: We appreciate this helpful suggestion. In the original manuscript, the sentence read:

> “The approach introduced by [18] tackles this issue by evaluating the influence of unmeasured confounding in scenarios involving binary exposure and outcomes.”

To improve clarity, we have revised it in the updated manuscript (page 10, lines 257–260 of the revised manuscript with track changes) to:

> “To address this issue, Kasza, Wolfe and Schuster developed an approach (the KWS framework) to evaluate the influence of unmeasured confounding in scenarios involving binary exposure and outcome [18].”

Methods

5. MICS data collection was done from Jan to June 2019 - It is important to acknowledge the presence of Covid 19 during data collection. How does this affect what is being observed by the study?

Author Response: Thank you for raising this point. We would like to clarify that the data used in our study were collected during January–June 2019, prior to the emergence of the COVID-19 pandemic.

The SARS-CoV-2 virus responsible for the COVID-19 pandemic first appeared in December 2019 in Wuhan, China. The World Health Organization declared COVID-19 as a Global Public Health Emergency

on 30 January 2020. In Bangladesh, the first confirmed cases were reported on 8 March 2020, well after the completion of the MICS 2019 survey [19].

Therefore, the COVID-19 pandemic did not overlap with the survey timeline and should not have influenced the data collection or the study results.

Consistency of terms

6. coc and mad vs CoC and MAD - rather stick to one way of writing these terms so that you do not lose the reader.

   Author Response: Thank you for pointing this out. We acknowledge this inconsistency in the earlier version of the manuscript. In the revised version, we now use "CoC" and "MAD" consistently throughout the text to ensure clarity and readability.

Reviewer 2

Thank you for the opportunity to review this manuscript. The authors provide an interesting methodology to evaluate the causal relationship between the maternal continuum of care and the child's minimum acceptable diet. The article is well written, concepts are well explained, and comprehensive analysis has been done. The methods section contains a thorough explanation of the variables included, and confounders that have been accounted for, and a detailed description of the analysis approach taken. The use of secondary data for this analysis is commendable.

Author Response: Thank you!

Introduction

1. Pg 2, line 16-22: Please rephrase, to make it clear that MDD previously represented the consumption of four out of seven groups, but is now classified as the consumption of five out of eight food groups.

   Author Response: Thank you for this helpful suggestion. We agree that the earlier version of the paragraph lacked clarity and reading flow in explaining the change in the definition of MDD. To improve the readability, we have revised the text to more clearly describe the transition from the original definition (four out of seven food groups) to the updated definition (five out of eight food groups).

   The previous version read as follows:

"MDD represents the consumption of four food groups among seven food groups to meet proper daily nutrition and for higher dietary quality. The seven food groups are grains, roots and tubers; legumes and nuts; dairy products; flesh foods (meat, fish, poultry and organ meats); eggs; vitamin-A rich fruits and vegetables; other fruits and vegetables [20]. In 2017, the WHO Technical Expert Advisory Group on Nutrition Monitoring (TEAM) updated its definition to include breast milk as a food group. As a result, MDD now refers to the consumption of at least five out of eight food groups by a child (MDD-8) [21]."

The revised version (page 2-3, lines 16-26 of the revised manuscript with track changes) now reads as follows:

"MDD was originally defined as the consumption of at least four out of seven food groups to ensure adequate daily nutrition and higher dietary quality. The seven food groups included grains, roots and tubers; legumes and nuts; dairy products (e.g., infant formula, milk, cheese, yogurt); flesh foods (meat, fish, poultry, and organ meats); eggs; vitamin A–rich fruits and vegetables; and other fruits and vegetables [20]. In 2017, the WHO Technical Expert Advisory Group on Nutrition Monitoring (TEAM) revised this definition to incorporate breast milk as an additional food group. Accordingly, MDD is now defined as the consumption of at least five out of eight food groups by a child [21]."

We believe this revision provides greater clarity and coherence in describing the change in MDD definitions.

2. Additionally, on pg 2, line 35-36 the definition "MDD requirement (consuming at least 4 out of 6 food groups, excluding dairy products)" seems to the explanation of MDD in the methods section (pg 4, line 120-124 where all 8 groups are included. Please clarify.

Author Response: Thank you for pointing this out. In the earlier version of the manuscript, the definitions of MDD, MMF, and MAD were presented in different sections, which could have created ambiguity and the impression of conflicting explanations, particularly regarding the MDD requirement for non-breastfed children. For example, we had previously written in the "Introduction" that "for non-breastfed children, MAD is satisfied when they meet the MDD requirement (consuming at least 4 out of 6 food groups, excluding dairy products), alongside the MMF and at least two milk feeds", while in the "Methods" section we described MDD as the consumption of foods from five out of eight food groups. We agree that this was unclear and may have caused confusion.

To address this, in the revised manuscript we have kept the definitions of MDD and MMF only in the "Introduction" (page 2-3, lines 12-52 of the revised manuscript with track changes), followed immediately by a clear definition of MAD that incorporates both MDD and MMF. Specifically, we now write:

"MAD incorporates both MDD and MMF to evaluate a child's nutritional adequacy. For breastfed children, MAD is achieved when both the MDD and MMF requirements are satisfied. For non-breastfed children, MAD is met when they fulfill the MDD and MMF requirements and additionally consume at least two milk feeds [1]"

In the "Methods" section, (page 7, lines 161-163 of the revised manuscript with track changes), we now only describe MAD (the outcome variable), coded as binary depending on whether the child met the previously defined MDD and MMF criteria, thereby avoiding redundant or inconsistent wording.

This revision removes the earlier ambiguity and ensures that the definitions of MDD, MMF, and MAD are fully aligned with current WHO guidelines, while also presenting them in a clearer and more coherent manner.

3. Pg 3 line 84: "Questions", not "question" – meaning of sentence is unclear, please rephrase

Author Response: Thank you for pointing out the issue with clarity and wording in this sentence. In the previous version, the line read:

"Question may arise about considering CoC as the exposure of MAD intake."

We agree that this phrasing was both grammatically incorrect and unclear. To improve readability, we have revised the sentence to (page 5, lines 103-106 of the revised manuscript with track changes):

"Potential concerns may arise regarding the causal pathway between mothers' receiving CoC and children's MAD intake, particularly with respect to how the temporal ordering of exposure and outcome can be inferred from a cross-sectional survey."

We hope this rephrasing resolves the earlier ambiguity and conveys the intended meaning more clearly.

Methods

4. Ethical considerations: Although mention is made that the primary study had ethical clearance, it is unclear that permission was granted by study participants for this data to be used for the purpose of this secondary data analysis, please clarify.

Author response: Thank you for raising this important point regarding ethical considerations. Our study used publicly available, anonymized secondary data from the MICS 2019 survey, which contains no personally identifiable information [17]. Therefore, no additional ethical approval was required for this analysis. To ensure transparency, we have revised the "Ethical Considerations and approval" subsection to state (page 7, lines 157-159 of the revised manuscript with track changes):

> "For the present study, no additional ethical approval was required because the analysis was based on publicly available, de-identified secondary data from the MICS 2019 survey."

This revision makes clear the ethical basis of our work.

5. There is repetition of how to classify MAD in the introduction, as well as the methods section. Although this is usually in the methods section, as other definitions are provided in the introduction, I suggest keeping the classification to the introduction to prevent repetition.

   Author Response: Thank you for this helpful suggestion. We have revised the manuscript so that the definitions of MAD and its components (MDD and MMF) are presented only in the "Introduction" (page 2-3, lines 12-50 of the revised manuscript with track changes). In the Methods section, we now only indicate how MAD was binary coded for analysis.

6. Figure 5: The figure heading states that the vertical dotted line represents an ATE value of 0, but it is plotted at 1.0 on the x-axis (which represents ATE according to the figure). Please clarify this.

   Author Response: Thank you for raising this point. Upon review, we have realized that the caption for Fig 5. incorrectly stated that the vertical dotted line represents an ATE value of 0. This was a typographical error. The vertical dotted line is actually plotted at 1.0 on the x-axis, which denotes no causal effect. We have corrected the caption in the revised manuscript (page 13 of the revised manuscript with track changes) to reflect this accurately.

7. Pg 8, Line 258: There is missing text, sentence ends with "framework introduced by". Please rephrase.

   Author Response: Thank you for pointing this out. In the earlier version of the manuscript, the sentence ended with "framework introduced by" without specifying the intended reference, resulting in incomplete text. We have corrected this in the revised manuscript (page 13, lines 313–315 of the revised manuscript with track changes) as follows:

   > "We also performed a sensitivity analysis to assess whether our observed effect estimates remained robust or were influenced by unmeasured confounding, applying the framework described in [22]."

   This revision clarifies the intended reference and ensures the sentence is now complete.

8. Pg 8, Line 261: Please replace "coc on mad" with "CoC on MAD"

Author Response: Thank you for this helpful suggestion. In the revised manuscript, we have replaced "coc on mad" with "CoC on MAD" (page 14, line 318 of the revised manuscript with track changes) to maintain consistency and proper use of abbreviations throughout the text.

Discussion

9. Given the findings of this study, and the resource-limited setting of Bangladesh, the discussion would be strengthened by a more robust examination of actions policymakers need to take to improve achievement of CoC.

   Author Response: We thank the reviewer for this thoughtful suggestion. In response, we have expanded the "Discussion" subsection to provide a more robust examination of plausible policy actions that can improve the achievement of CoC in Bangladesh (page 15-16, lines 352-380 of the revised manuscript with track changes). Specifically, we describe strategies targeting both health system capacity and broader social determinants of maternal care. These include expanding access to antenatal care in underserved areas through mobile clinics, strengthening the role and training of community health workers, and improving maternal referral systems between local clinics and hospitals with skilled birth attendants. Additionally, we highlight social and economic interventions such as women's empowerment initiatives, targeted health promotion programs, conditional cash transfers, microcredit, and livelihood support, alongside integrated home-to-hospital healthcare systems and incentives for rural health professionals.

   Each of these recommendations is supported by existing literature [5–12]. Collectively, these measures could substantially enhance CoC completion and, in turn, improve maternal and child health outcomes in resource-limited settings.

10. Pg 8, line 290: I would argue that the statement "Our findings suggest that comprehensive maternal care plays a critical role in ensuring that children receive appropriate nutrition during the first two years of life", should be expanded to include the first 1000 days (from conception to the age of two); given that CoC encompasses the antenatal period too.

    Author Response: We thank the reviewer for this thoughtful suggestion. We fully agree that the first 1,000 days (i.e, the 270 days in utero and the first two years after birth) constitute a critical period for child nutrition and development, as highlighted by global guidelines.

    However, in this study we have focused specifically on the effect of CoC on MAD, which is an indicator of child nutrition from complementary meals alongside breastmilk. Therefore, the findings of this study can be most appropriately generalized to the first two years of life since birth. Extending the statement

to the first 1,000 days would also encompass maternal nutrition during pregnancy, which was outside the scope of the analysis in this study.

Accordingly, we have revised the sentence to read (page 15, lines 347-349 of the revised manuscript with track changes):

> "Our findings suggest that comprehensive maternal care plays a critical role in ensuring that children receive appropriate nutrition from complementary meals alongside breastmilk during the first two years of life, a crucial period for child development."

We believe this revision improves the clarity and precision of the statement while aligning it more closely with the scope of our study.

References

[1] WHO, UNICEF. Indicators for assessing infant and young child feeding practices: definitions and measurement methods [Internet]. World Health Organization; 2021 [cited 10 September 2025]. Available from: https://www.who.int/publications/i/item/9789240018389.

[2] Tamang TM. Factors associated with completion of continuum of Care for Maternal Health in Nepal. In: IUSSP XXVIII International Population Conference. Cape Town, South Africa: Statistics South Africa (Stats SA); 2017. Available from: https://www.academia.edu/70631689/Factors_Associated_with_Completion_of_Continuum_of_Care_for_Maternal_Health_in_Nepal.

[3] Alem AZ, Shitu K, Alamneh TS. Coverage and factors associated with completion of continuum of care for maternal health in sub-Saharan Africa: a multicountry analysis. BMC Pregnancy and Childbirth. 2022 May;22(1):422. doi:10.1186/s12884-022-04757-1.

[4] WHO I, FIGO. Making pregnancy safer: the critical role of the skilled attendant: a joint statement by WHO, ICM and FIGO [Internet]. World Health Organization; 2004 [cited 10 September 2025]. Available from: https://www.who.int/publications/i/item/9241591692.

[5] Bawadi HA, Abu Abed AS, Al-Hamdan ZM, Alzubi SM. Receiving antenatal care via mobile clinic: Lived experiences of Jordanian mothers. International Journal of Nursing Sciences. 2023 Apr;10(2):230-7. doi:10.1016/j.ijnss.2023.03.005.

[6] Scharff D, Enard KR, Tao D, Strand G, Yakubu R, Cope V. Community Health Worker Impact on Knowledge, Antenatal Care, And Birth Outcomes: A Systematic Review. Maternal and Child Health Journal. 2022 Jan;26(1):79-101. doi:10.1007/s10995-021-03299-w.

[7] Mengist B, Semahegn A, Yibabie S, Amsalu B, Tura AK. Barriers to proper maternal referral system in selected health facilities in Eastern Ethiopia: a qualitative study. BMC Health Services Research. 2024 Mar;24(1):376. doi:10.1186/s12913-024-10825-3.

[8] Olaniran A, Madaj B, Bar-Zev S, van den Broek N. The roles of community health workers who provide maternal and newborn health services: case studies from Africa and Asia. BMJ Global Health. 2019 Aug;4(4):e001388. doi:10.1136/bmjgh-2019-001388.

[9] Rahman R, Rahman M, Haque SE. Empowerment dimensions and their relationship with continuum care for maternal health in Bangladesh. Scientific Reports. 2021 Sep;11(1):18760. doi:10.1038/s41598-021-98181-8.

[10] Schuler SR, Hashemi SM. Credit Programs, Women's Empowerment, and Contraceptive use in Rural Bangladesh. Studies in Family Planning. 1994 Mar-Apr;25(2):65-76. doi:10.2307/2138085.

[11] Mahmood SS, Amos M, Hoque S, Mia MN, Chowdhury AH, Hanifi SMA, et al. Does healthcare voucher provision improve utilisation in the continuum of maternal care for poor pregnant women? Experience from Bangladesh. Global Health Action. 2019 Dec;12(1):1701324. doi:10.1080/16549716.2019.1701324.

[12] Edwards C, Saha S. From home to hospital, a continuum of care: making progress towards Millennium Development Goals 4 and 5 in rural Bangladesh. BJOG: An International Journal of Obstetrics & Gynaecology. 2011 Sep;118(s2):88-92. doi:10.1111/j.1471-0528.2011.03116.x.

[13] RAPHAEL K. Recall Bias: A Proposal for Assessment and Control. International Journal of Epidemiology. 1987 Jun;16(2):167-70. doi:10.1093/ije/16.2.167.

[14] Wang X, Cheng Z. Cross-Sectional Studies: Strengths, Weaknesses, and Recommendations. Chest. 2020 Jul;158(1, Supplement):S65-71. doi:10.1016/j.chest.2020.03.012.

[15] Li F, Zaslavsky AM, Landrum MB. Propensity score weighting with multilevel data. Statistics in Medicine. 2013 Aug;32(19):3373-87. doi:10.1002/sim.5786.

[16] Tulchinsky TH, Varavikova EA. Chapter 3 - Measuring, Monitoring, and Evaluating the Health of a Population. In: Tulchinsky TH, Varavikova EA, editors. The New Public Health. 3rd ed. San Diego: Academic Press; 2014. p. 91-147. doi:10.1016/B978-0-12-415766-8.00003-3.

[17] BBS, UNICEF. Progotir Pathey, Bangladesh Multiple Indicator Cluster Survey 2019, Survey Findings Report. Dhaka, Bangladesh: Bangladesh Bureau of Statistics (BBS) and UNICEF Bangladesh; 2019. Available from: https://www.unicef.org/bangladesh/media/3281/file/Bangladesh%202019%20MICS%20Report_English.pdf.

[18] Kasza J, Wolfe R, Schuster T. Assessing the impact of unmeasured confounding for binary outcomes using confounding functions. International Journal of Epidemiology. 2017 Aug;46(4):1303-11. doi:10.1093/ije/dyx023.

[19] Ahmed SM, Khanam M, Shuchi NS. COVID-19 pandemic in Bangladesh: A scoping review of governance issues affecting response in public sector. Public Health in Practice. 2024 Jun;7:100457. doi:10.1016/j.puhip.2023.100457.

[20] WHO, USAID, AED, DAVIS U, UNICEF. Indicators for assessing infant and young child feeding practices: Part 1: Definitions: Conclusions of a consensus meeting held 6-8 November 2007 in Washington DC, USA [Internet]. World Health Organization; 2008 [cited 10 September 2025]. Available from: https://www.who.int/publications/i/item/9789241596664.

[21] WHO. Global Nutrition Monitoring Framework: operational guidance for tracking progress in meeting targets for 2025 [Internet]. World Health Organization; 2017 [cited 10 September 2025]. Available from: https://www.who.int/publications/i/item/9789241513609.

[22] Ciocănea-Teodorescu I, Sjölander A. A cautionary note on a recently proposed sensitivity analysis for unmeasured confounding. International Journal of Epidemiology. 2021 Jun;50(3):711-6. doi:10.1093/ije/dyaa258.
